# Vacunas, equidad y soberanía: ciencia desde Colombia para el mundo

**DOI:** 10.7705/biomedica.8030

**Published:** 2025-05-30

**Authors:** Natalia Muñoz-Durango, Juan Pablo Hernández-Ortiz, Jorge E. Osorio

**Affiliations:** 1 Directora de Proyectos Especiales, VaxThera S.A.S., Medellín, Colombia VaxThera S.A.S. VaxThera S.A.S. Medellín Colombia; 2 Profesor, Universidad de Nacional de Colombia y director, Laboratorio Genómico OneHealth, Universidad Nacional de Colombia, sede Medellín; COO, VaxThera S.A.S., Medellín, Colombia Universidad de Nacional de Colombia Universidad de Nacional de Colombia Medellín Colombia; 3 Professor, Department of Pathobiological Sciences, University of Wisconsin School of Veterinary Medicine, Madison, WI; director, Global Health Institute, University of Wisconsin- Madison, Madison, WI, y CEO, VaxThera S.A.S., Medellín, Colombia University of Wisconsin School of Veterinary Medicine University of Wisconsin School of Veterinary Medicine Medellín Colombia

Las vacunas han sido uno de los avances más significativos en la historia de la salud pública. Desde la erradicación de la viruela hasta la reducción drástica de enfermedades, como la poliomielitis y el sarampión, las vacunas han salvado 154 millones de vidas en los últimos cincuenta años y han mejorado la calidad de vida de las personas en todos los rincones del planeta. Sin embargo, a medida que avanzamos hacia un futuro en el cual las vacunas jugarán un papel aún más crucial, enfrentamos desafíos significativos en su disponibilidad, distribución, acceso equitativo y actualización de sus versiones, especialmente en los países de bajos recursos ^1^.

El impacto transformador de las vacunas en la salud global se ha manifestado en múltiples frentes. Han permitido prevenir epidemias devastadoras y han mejorado significativamente la salud materna e infantil. La inmunización durante el embarazo, por ejemplo, protege tanto a la madre como al recién nacido frente a enfermedades graves. En ese mismo sentido, vacunas como la del rotavirus han contribuido a disminuir de manera considerable la mortalidad infantil asociada con diarreas agudas, una de las principales causas de muerte en niños menores de cinco años ^2^.

No cabe duda de que los programas nacionales de inmunización han generado importantes beneficios en poblaciones priorizadas como la de niños y la de adultos mayores. No obstante, los cambios demográficos de nuestra sociedad, con una población cada vez más envejecida, exigen una evolución del enfoque: es momento de implementar una vacunación con perspectiva de curso de vida. Esto implica fortalecer la cultura de inmunización en adultos, no solo en grupos de riesgo. Las campañas masivas que protejan a la población laboralmente activa contra infecciones respiratorias, como la influenza o la neumonía neumocócica, así como esquemas más inclusivos de vacunación contra el virus del papiloma humano en géneros y edades tradicionalmente no cubiertos por los programas gubernamentales (como la población entre los 18 y los 46 años), son medidas urgentes y necesarias ^1^.

A pesar de los logros, la infraestructura para garantizar una vacunación efectiva continúa siendo un reto. En muchas regiones, especialmente en las zonas rurales, la cadena de frío es difícil de mantener, y el almacenamiento y la administración de productos biológicos se ven comprometidos por carencias logísticas y de talento humano capacitado. Además, la complejidad de las redes globales de suministro, que abarcan desde materias primas hasta mecanismos de distribución, expone a los sistemas de salud a vulnerabilidades en tiempos de crisis o escasez.

La pandemia del COVID-19 puso en evidencia de manera contundente estas desigualdades. Mientras los países con mayores recursos garantizaban rápidamente dosis para su población, muchos de los países en desarrollo quedaban rezagados, enfrentando barreras económicas, tecnológicas y diplomáticas. Esta desigualdad no solo refleja brechas de equidad, sino que compromete la salud global: los virus no conocen fronteras.

Nuestra región no ha sido ajena a estas tensiones. Brotes como los de dengue, chikunguña, Zika o, más recientemente, fiebre amarilla en Colombia, revelan lo frágil de nuestra autonomía sanitaria. Aunque Latinoamérica cuenta con centros de producción de vacunas en México, Brasil, Argentina y Cuba, ninguno de estos países alcanza una autosuficiencia real en el abastecimiento de vacunas para sus planes ampliados de inmunización. En conjunto, apenas cubrirían cerca del 40 % de la demanda regional ^3^, dejando una dependencia estructural preocupante.

Un caso emblemático fue la epidemia por el virus del Zika entre el 2015 y el 2016, que afectó a más de 100.000 personas en Colombia ^4^ y alrededor de 400.000 en Latinoamérica ^5^. En Colombia, se registraron 18.177 mujeres gestantes infectadas y, al menos, 248 niños nacidos con síndrome congénito del Zika ^6^. En estudios posteriores, se encontró que cerca del 2 % de los hijos de madres infectadas presentaba deficiencias cerebrales o auditivas ^7^. Las pérdidas económicas asociadas con esta epidemia se estiman entre los 7 y los 18 billones de dólares para los sistemas de salud de la región ^8^, sin contar el costo social y el emocional. Si bien hasta el momento no hay vacunas disponibles para humanos contra el virus del Zika, esta epidemia expuso la necesidad de enfocar los esfuerzos en el desarrollo de soluciones para las enfermedades que aquejan a nuestras poblaciones, y que apuntan a la investigación y el desarrollo como pilares fundamentales para lograrlo.

Más recientemente, en abril de 2025, el gobierno colombiano declaró la emergencia sanitaria por un brote de fiebre amarilla que ya ha cobrado la vida de más de 30 personas en zonas rurales de nueve departamentos ^9^. Aunque contra esta enfermedad sí se cuenta con una vacuna efectiva, la escasez global y las dificultades en la distribución han impedido una respuesta rápida y efectiva, evidenciando nuevamente la importancia de contar con capacidad local de producción y distribución de productos biológicos.

Ante esta realidad, Colombia vive un momento histórico. Existe una confluencia favorable de voluntad política, compromiso académico y acción empresarial, para recuperar y fortalecer la soberanía sanitaria. Una de las iniciativas que representa esta visión de soberanía sanitaria es VaxThera (www.vaxthera.com), empresa biotecnológica colombiana. En menos de cuatro años, la compañía ha avanzado significativamente en distintos frentes estratégicos, como la construcción de una planta de manufactura de vacunas humanas en Rionegro (Antioquia), que se proyecta como una de las más modernas de la región y cuya certificación en buenas prácticas de manufactura por parte del Instituto Nacional de Vigilancia de Medicamentos y Alimentos (Invima) está prevista para el 2025. Además, cuenta con un laboratorio de investigación y desarrollo en Medellín, que opera desde el 2023, dedicado al diseño y la evaluación de vacunas contra enfermedades infecciosas emergentes, tropicales, y contra aquellas de potencial pandémico, con énfasis en las que afectan a nuestra región, como el dengue, la fiebre amarilla y el Zika.

En colaboración con grupos de investigación nacionales e internacionales, VaxThera ha combinado la vigilancia genómica con ciencia de frontera para responder con agilidad ante los agentes patógenos que circulan en Colombia y en Latinoamérica. Este conocimiento no solo alimenta las plataformas tecnológicas, como las vacunas de ARN mensajero y vectores virales, sino que, además, permite desarrollar soluciones que puedan ser compartidas mediante mecanismos de cooperación científica sur-sur y sur-norte, proyectando ciencia desarrollada en Colombia para el mundo. Este esfuerzo responde directamente a las políticas de reindustrialización del sector salud del país y requiere del apoyo de todos los actores del sistema para consolidar una infraestructura sólida y sostenible.

El acceso a la salud no debe ser determinado por la geografía ni por el nivel de ingreso. Por ello, la apuesta de VaxThera es clara y compartida entre los diversos actores:

*“[...] trabajaremos para convertir a Colombia en un referente regional en producción, innovación y acceso a vacunas y tratamientos biotecnológicos, con ciencia y talento local, pero con impacto global […]”.*

El futuro de las vacunas, y de nuestra salud colectiva, depende de nuestra capacidad de trabajar en conjunto: desde el fortalecimiento de capacidades de regulación hasta la formación de talento humano, la inversión en infraestructura y la cooperación científica regional. Solo así podremos garantizar que cada persona, sin importar su lugar de origen, tenga acceso oportuno a tecnologías que salvan vidas.

Las vacunas han transformado el mundo. Ahora, nos corresponde transformar el acceso a ellas.
